# Improving *de novo* Molecule Generation by Embedding LSTM and Attention Mechanism in CycleGAN

**DOI:** 10.3389/fgene.2021.709500

**Published:** 2021-08-05

**Authors:** Feng Wang, Xiaochen Feng, Xiao Guo, Lei Xu, Liangxu Xie, Shan Chang

**Affiliations:** ^1^Changzhou University Huaide College, Taizhou, China; ^2^School of Computer Science and Artificial Intelligence, Aliyun School of Big Data, Changzhou University, Changzhou, China; ^3^Institute of Bioinformatics and Medical Engineering, Jiangsu University of Technology, Changzhou, China

**Keywords:** LSTM, attention mechanism, Mol-CycleGAN, head-to-tail feature fusion, LA-CycleGAN

## Abstract

The application of deep learning in the field of drug discovery brings the development and expansion of molecular generative models along with new challenges in this field. One of challenges in *de novo* molecular generation is how to produce new reasonable molecules with desired pharmacological, physical, and chemical properties. To improve the similarity between the generated molecule and the starting molecule, we propose a new molecule generation model by embedding Long Short-Term Memory (LSTM) and Attention mechanism in CycleGAN architecture, LA-CycleGAN. The network layer of the generator in CycleGAN is fused head and tail to improve the similarity of the generated structure. The embedded LSTM and Attention mechanism can overcome long-term dependency problems in treating the normally used SMILES input. From our quantitative evaluation, we present that LA-CycleGAN expands the chemical space of the molecules and improves the ability of structure conversion. The generated molecules are highly similar to the starting compound structures while obtaining expected molecular properties during cycle generative adversarial network learning, which comprehensively improves the performance of the generative model.

## Introduction

Computer-aided drug Design (CADD) promotes the speed of drug discovery (Macalino et al., 2015). Beyond the traditional CADD, artificial intelligence (AI) is widely used in the process of new drug screening and optimization. AI is realized by using various kinds of machine learning or deep learning (DL) algorithms (Goodfellow et al., 2016; Lavecchia, 2019). Among different methods, DL method generally trains a large amount of sample data through neural networks to learn the molecular structure of the sample. Different from the traditional similar ligand searching, the DL model obtains the characteristic information and general rules from learning prior knowledge during the training process. In the field of DL, generative models can generate novel compounds effectively with the desired properties, which would reduce the cost of drug discovery (Jing et al., 2018; Wainberg et al., 2018). Drug discovery and design (Muegge et al., 2017; Agrawal, 2018) use the knowledge of available biomedicine (Mamoshina et al., 2016; Tang et al., 2019) to define the parameters and indicators that required by each drug molecule in the following processes: (1) Selection and confirmation of drug targets; (2) Discovery of seed compound (Hit); (3) Discovery and optimization of lead compounds (Lead); (4) Discovery of Candidate. Among the processes, molecule generation (Xu et al., 2019) emergences as new tool in the hit-to-lead and lead optimization phases of drug discovery.

The generation model (Jensen, 2019; Walters and Murcko, 2020) is used to generate new molecules with similar molecular activity to the trained compounds, and to learn the distribution characteristics of data by using unsupervised learning (Radford et al., 2015). The generative models normally use SMILES (Weininger, 1988; Öztürk et al., 2016) grammar based on ASCII characters and molecular graphs based on Graph (Li et al., 2018; Lim et al., 2020) to describe molecules at the atomic level. For example, recurrent neural network (RNN) can generate a larger chemical space than the training set by using SMILES grammar to input a small part of molecular data set (Arús-Pous et al., 2019). Based on deep learning methods, several deep generative models have been proposed. Deep generative models are roughly divided into four categories: (1) Based on the Auto Encoder (AE) model used in semi-supervised learning and unsupervised learning; (2) Generative Adversarial Networks (GAN)(Goodfellow et al., 2014) model, which is composed of two architectures: generator and discriminator, and one-way generation that confronts each other during the training process; (3) Model based on RNN (Méndez-Lucio et al., 2020); (4) Hybrid model based on the combination of deep generative model and reinforcement learning (RL) (Grisoni et al., 2020).

Different architectures are ongoing developed to generate more realistic molecules (Sarmad et al., 2019). Character-level recurrent neural network (CharRNN) (Segler et al., 2018) is trained on the SMILES molecular database. CharRNN is a chemical language model based on SMILES grammar specifications. It uses Maximum Likelihood Estimation (MLE) to optimize model parameters and improve the structural similarity of the generated molecules. CharRNN can generate molecules with new pharmacological properties. Variational Autoencoder (VAE) (Gómez-Bombarelli et al., 2018; Simonovsky and Komodakis, 2018) is a kind of “encoder-decoder” architecture. It proposes a new method based on the continuous coding of molecules to explore the chemical space. This model map high-dimensional data to latent space, and perform a new search based on directional gradients in chemical space. The Adversarial Autoencoder (AAE) (Makhzani et al., 2015; Kadurin et al., 2017) combines the ideas of adversarial training in VAE and GAN for the first time. Junction Tree Variational Autoencoder (JTN-VAE) (Jin et al., 2018) directly uses molecular graph expression. JTN-VAE alternatively generates linear SMILES strings to complete the task of molecule generation. First, the chemical substructure on the generated tree object is automatically decomposed, and the substructure on the training set is extracted. Then, these substructures are combined into a molecular map. Based on GAN, LatentGAN (Prykhodko et al., 2019) combines an autoencoder and a generative adversarial to carry out new molecular designs. CycleGAN (Zhu et al., 2017) combines two symmetrical GANs into a ring network, which has two generators and two discriminators to perform two data conversions. The Mol-CycleGAN model (Maziarka et al., 2020) extends CycleGAN to the JT-VAE framework to ensure that the generated compounds are always effective. The original molecular data set is entered into the “encoder-decoder” architecture in order to obtain a novel compound that is similar to the original molecular structure with the required pharmacological properties. Mol-CycleGAN does not use SMILES grammar and atomic ordering, which is a heavy training burden. The decoding method selected by the model is combined with the JT-VAE based on the Graph form, so that the generated molecule is always effective.

In this article, we proposed LA-CycleGAN by embedding CycleGAN architecture with long and short-term memory module (LSTM) (Mao et al., 2017) and Attention mechanism. We optimized the generator and discriminator of CycleGAN with the aim to improve the training performance of the network model and expand the chemical space. LSTM and Attention mechanism are used to solve long-term dependency problems when treating SMILES grammar inputs. The network layer of the generator of the confrontation network is merged end to end. Least Squares Generative Adversarial Network (LSGAN) (Yasonik, 2020) is used to learn the corresponding transformation by minimizing the loss. The results show that the optimized model improves the ability of structural transformation. The structure similarity between the generating molecule and the starting molecule is increased, which should satisfy the specific requirements for drug production.

## Materials and Methods

### LSTM

**FIGURE 1:**
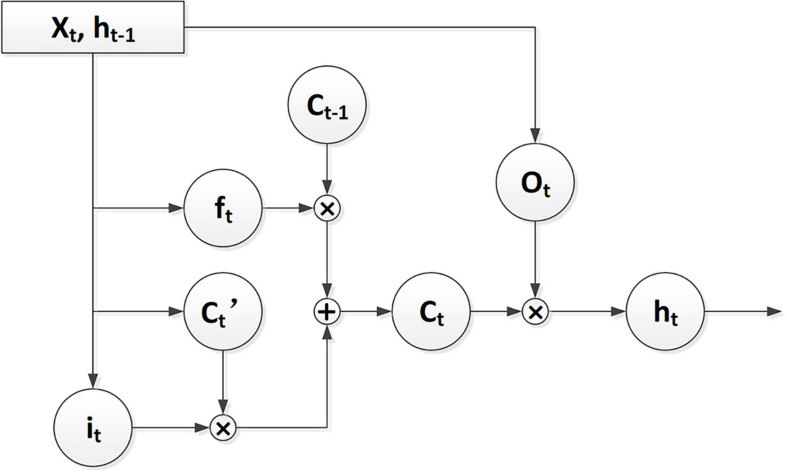
The flow chart of LSTM architecture.

Long Short-Term Memory (LSTM) is a network structure extended from the RNN. This structure is mainly used to solve the problem of gradient disappearance and gradient explosion in the long sequence training process of SMILES grammar. The LSTM structure is shown in Figure 1.

Long Short-Term Memory (LSTM) can control the transmission state through three gating states. With the gating states, LSTM can process the characterization of various sequence lengths and perform globalization processing. Therefore, the SMILES can still have a better characterization when the SMILES type is longer. *f*_*t*_ stands for forget gate, whose structure purposefully deletes or adds information to the input sequence, and effectively handles the generation of molecular structures described in SMILES form. *i*_*t*_ stands for input gate used to update the unit state and determine whether the information is stored. *C*_*t*_ represents the output used by the output gate to control. As shown in Eq. (1).

(1) ht=σ(Wo⋅[ht-1,x]t+bo)⏟ot*tanh(σ(Wf⋅[ht-1,x+tbf)⏟ft]*Ct-1+σ(Wi⋅[ht-1,x]t+bi)⏟it*tanh(WC⋅[ht-1,x]t+bC)⏟Ct′))⏟Ct

*h*_*t–1*_ represents the output of the previous state and *x*_*t*_ represents the input to the current state. σ represents the activation function of sigmoid on the gate and tanh represents the activation function on the state and output. *W* represents the weight, *b* represents the bias, and *h*_*t*_ represents the output of the current state. ⊗ is unit times, and ⊕ is unit plus.

### Attention Mechanism

The Attention mechanism (Vaswani et al., 2017) also handles the chemical structural formula sequence globally. The vector generated by the LSTM module cannot fully represent the information of the entire sequence. The attention mechanism can retain the intermediate output results of the LSTM encoder on the input sequence. It is used to supplement the SMILES information lost by LSTM and perform secondary learning on the features that have not been learned. The process of re-modeling global information can effectively improve the performance of the model. The Attention mechanism is similar to the task mechanism of LSTM, which overcomes the problem of information loss in the feature expression process. It effectively focuses on the output results of the encoder according to the model target. The Attention mechanism is put at the bottom of the context vector insertion layer. The output vector of the LSTM is used as the input of the Attention mechanism, which is used to calculate the vector probability distribution of the feature. The method can capture the global information of the chemical formula structure. Attention mechanism solves the long-distance dependence of input sequence in RNN. The function of Attention mechanism is as shown in Eq. (2).

(2) $$Attention\left(Query,Key,Value\right)=soft\text{max}\left(\frac{Query⊙Key^{T}}{\sqrt{d}}\right)*Value$$

*Query* represents the current molecule fragment, *Key* represents each molecule, and *d* is the vector dimension of *query* and *key*. *Softmax* is used to make the probability distribution of the result. *Value* represents the current molecule, and re-obtain important information. The attention mechanism can make deep neural network interpretable by capturing the important features. Attention mechanism assigns attention scores for the input features. The attention score can be interpreted as the importance of the feature, which will filter out the useless molecular feature information.

### Model Architecture of LA-CycleGAN

During the optimization process of the model generated by Mol-CycleGAN, LSTM neural network and Attention mechanism are embedded in the framework of CycleGAN. The internal network layers of the generator and the discriminator are merged end to end. The first and last layers of the internal network layer are merged for the generator and the discriminator. During the training process, we use LSGAN loss and Batch Normalization (BN) (Ioffe and Szegedy, 2015). Mol-CycleGAN directly uses JT-VAE to generate latent vectors, which is convenient for molecular graphic expression. The purpose of CycleGAN is to learn the method from the original molecular data domain X to the target molecular data domain Y.

Through training mapping _*G:X→Y*_ and reverse mapping _*F:Y→X*_, _*F(G(X))≈X*_, and _*G(F(Y))≈Y*_ are established at the same time. In order to prevent generators G and F from stopping the conversion function after generating data, we use cycle consistency loss as an incentive, as in Eq. (3).

(3) $$L_{cyc}\left(G,F\right)=E_{x∼Pdata\left(x\right)}\left[{||F\left(G\left(\text{x}\right)\right)-x||}_{1}\right]+E_{y∼Pdata\left(y\right)}\left[{||G\left(F\left(y\right)\right)-y||}_{1}\right]$$

In order to prevent overfitting between input and output, the identity mapping loss is used to ensure that the generated molecule is close to the starting molecule, as in Eq. (4).

(4) $$L_{identity}\left(G,F\right)=E_{y∼Pdata\left(y\right)}\left[{||F\left(y\right)-y||}_{1}\right]+E_{x∼Pdata\left(x\right)}\left[{||G\left(x\right)-x||}_{1}\right]$$

In order to ensure that the two generators can achieve mutual inversion, the overall loss function is used, where λ_1_ = 0.4, λ_2_ = 0.15, as in Eq. (5).

(5) $$L\left(G,F,D_{X},D_{Y}\right)=L_{GAN}\left(G,D_{Y},Y,X\right)+L_{GAN}\left(F,D_{X},Y,X\right)+\lambda_{1}L_{GAN}\left(G,D_{Y},Y,X\right)+\lambda_{2}L_{identity}\left(G,F\right)$$

Where *Dx* is used to distinguish between *X* and *F*(*Y*), and *D*_*Y*_ is used to distinguish between *Y* and *G*(*X*). The two generators simultaneously carry out the reverse process of mutual fight against between optimization reduction and optimization increase. The overall loss function of the state is optimized according to Eq. (6).

(6) $$G*,F*={\text{argmin}}_{G,F}{\text{max}}_{D_{X},D_{Y}}L\left(G,F,D_{X},D_{Y}\right)$$

LS-GAN’s confrontation loss is introduced, as in Eq. (7).

(7) $$L_{cyc}\left(G,D_{Y},X,Y\right)=\frac{1}{2}E_{x∼Pdata\left(x\right)}\left[{\left(D_{Y}\left(G\left(X\right)\right)\right)}^{2}\right]+\frac{1}{2}E_{y∼Pdata\left(y\right)}\left[{\left(D_{Y}\left(\text{y}\right)-1\right)}^{2}\right]$$

The main idea of the molecular optimization method is to obtain molecular descriptors, which make the generated chemical molecules easier to synthesize and generate molecules similar to the original molecules.

### Workflow

**FIGURE 2:**
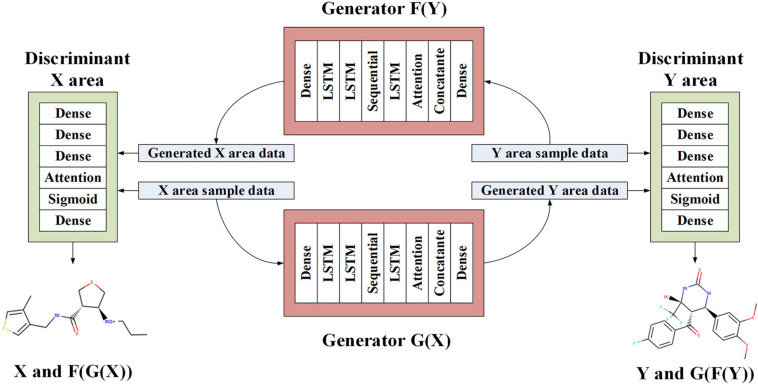
The proposed architecture of LA-CycleGAN. LSTM with Attention mechanism are embedded in the generators.

As shown in Figure 2, GAN also generates discrete sequences when training potential chemical spaces. Then, the encoder can force the latent space to generate some continuously distributed sequences when training on the molecular data set. We construct a self-encoder in the generator of CycleGAN, and use the input data sequence as the learning target for characterization learning. The generator is composed of three components: encoder, converter, and decoder. The encoder is responsible for converting the SMILES string into a digital representation feature. The converter performs information conversion. The decoder reconstructs the features and obtains a new SMILES string. An encoded SMILES string is input into an LSTM model with 56 hidden neurons, which is constructed as a single-layer network. The encoder obtains the characteristic information and distribution law of the input original sequence data through LSTM. The vector output by the encoder through multiple feature combination is input to the converter constructed by the linear structure Sequential model. The latent vector output by the encoder is input to the converter. The converter plays a transitional role, converting various molecules from the source domain to the target domain, avoiding information loss. The LSTM module acts as a decoder. The vector of each output vector table of the decoder unit is restored to the size of the starting vector, and regain the characteristic information of low-level molecules. Then, the decoded molecules still retain the structure or characteristics of the original molecular data, and are converted into samples in the target domain.

We map the output vector of the decoder to the Attention mechanism, perform learning mapping on the vector output from the encoder and then calculate the distribution probability. The output feature information is fused into the layer fusion module to ensure that each original molecule input can be directly input to the final fully connected layer. In LA-CycleGAN, the use of head-to-tail feature fusion plays an important role in improving the similarity of molecular generation. In the process of generating, the low-level feature information at the head is accepted by the high-level feature information at the tail. By using the feature fusion, a thicker feature can be obtained from splicing the channel dimensions of the features. Therefore, the generated molecular dataset is closer to the molecules of the original data domain. The original feature information is retained, and the deviation between the generated sample and the original sample is reduced. The original molecular features remain in the generated molecular features, resulting in an effective molecular structure.

The discriminator inputs the original SMILES data set and the generated data together. The discriminator is composed of multiple dense layers to extract feature vectors from the data. In order to determine whether these features belong to a specific category. The output feature vector of the Attention mechanism is processed by the feedforward network layer with sigmoid activation, and the probability of sampling each character of the known character set in the data set is feedback. The last layer of the discriminator network is the dense layer and produce one-dimensional output. It is used to realize the judgment of the similarity difference of the generated molecules to continue the training of the discriminator. The network structure parameters of the new model are shown in Table 1.

**TABLE 1:** LA-CycleGAN network layer structure parameters.

Network layer structure (generator)	Units	Network layer structure (Discriminator)	Units
Input	56	Input	56
Dense	56	Dense	56
LSTM	56	Dense	28
LSTM	28	Dense	56
LSTM	56	Attention	56, 64, 1
Attention	56, 64, 1	activation	Sigmoid
concatenate	57	Dense	1
Dense	56	–	–

### Data Set

ZINC database (Sterling and Irwin, 2015) is a molecule structure database. The deep generative model requires training on a large amount of data to be able to learn patterns that can generalize and generate new molecules. Therefore, a molecular data set is extracted from the ZINC database containing 250,000 drugs. The chemical characteristics of drug molecules are generally defined by FeatureType and FeatureFamily. Each FeatureType uses smarts expressions to describe the mode and attributes of the molecule, and summarize and characterize the molecular structure from different degrees.

Molecules with the same activity generally have common chemical characteristics. FeatureFamily classifies features as a whole to achieve the matching effect of pharmacophore (Nastase et al., 2019), which is an effective way to determine whether a molecule has a certain type of pharmacodynamic characteristics. It can assist the structural design of drug molecules. The pharmacophore model is a model based on the characteristic elements of pharmacodynamics. Pharmacophore includes Aliphatic Rings, Aromatic Rings, Hydrogen-Bonding Acceptor (HBA), Hydrogen-Bond Donor (HBD), and other characteristic elements, which can test the ability of molecular structure transformation. The characteristic elements of the pharmacophore are incorporated into one of the standards for the generation of compound molecules, which can effectively avoid the generation of molecular structures with large errors in similar compounds. The new type of compound generated is contrary to the design requirements. Then, we select X and Y with different structure distributions, and test whether our model can learn transformation rules and apply them to molecules that the model has not seen before. According to the characteristics of the pharmacophore elements, the datasets with different features are divided as follows:

• Aliphatic Rings: Aliphatic Ring compounds refer to the hydrocarbon group in the molecule containing a carbocyclic ring, and this carbocyclic ring can be saturated and unsaturated. The molecule in X has exactly 1 alicyclic ring, while the molecule in Y has 2 or 3 Aliphatic Rings.
• Aromatic Rings: The molecule in X has exactly 2 Aromatic Rings, while the molecule in Y has 1, 3, or 4 Aromatic Rings.
• Hydrogen bond acceptor (HBA): The electronegative atom is the hydrogen acceptor. The molecule in X has 1 hydrogen bond, and the molecule in Y has 2–3 hydrogen bond acceptors.
• Hydrogen bond donor (HBD): The molecule in X is a hydrogen bond, and the molecule in Y is 2, 3, or 4 hydrogen bond donors.

### Performance Evaluation Index

We use the evaluation indicators provided by MOSES (Polykovskiy et al., 2020) to evaluate the generated molecules. The generative model is evaluated by comparing the fragment similarity, scaffold similarity, nearest neighbor similarity, Tanimoto coefficient, Fréchet ChemNet Distance and internal diversity of the generated set *G*. **Valid** judges the generated SMILES string and checks the consistency of the valence and chemical bond of the generated molecule. **Filters** are part of the generated molecules, which pass the filters applied during the construction of the dataset to remove molecules containing charged atoms. **Novelty** is the proportion of generated molecules that do not appear in the training set, and low novelty represents overfitting. **Success rate** represents the success rate of molecular structure transformation. **Uniqueness** checks whether the model will collapse and only a few typical molecules are generated. **Non-identity** is the score when the generated molecule is different from the starting molecule.

**Fragment similarity (Frag)** is defined as the cosine distance between fragment frequency vectors, such as Eq. (8).

(8) $$Frag\left(G,R\right)=1-\text{cos}\left(F_{G},F_{R}\right)$$

For the fragment frequency vectors *F*_*G*_ and *F*_*R*_ of molecule *G* and molecule *R*, the size is equal to the vocabulary of all chemical fragments in the data set. The corresponding molecular element indicates the frequency of the corresponding fragment in the molecular set. This metric shows the similarity of the two groups of molecules at the chemical fragment level Degree and distance definition.

**Nearest neighbor similarity (SNN)** is the average Tanimoto similarity between the generated molecule and the nearest molecule in the test set, as in the Eq. (9).

(9) $$SNN\left(G,R\right)=\frac{1}{\left|G\right|}\sum_{m_{G}\in G}\max_{m_{R}\in R}T\left(m_{G},m_{R}\right)$$

The chemical structure encoded in the fingerprint, the nearest neighbor molecule *m*_*R*_ from the test set *R* and the molecule *m*_*G*_ in the generating set *G*.

**Internal Diversity (*IntDiv*_*p*_)** is the average pair-wise similarity of generated molecules, which is used to evaluate the chemical diversity in the generating set, as shown in Eq. (10).

(10) $$IntDiv_{p}\left(G\right)=1-\sqrt[{P}]{\frac{1}{{\left|G\right|}^{2}}\sum_{m_{1},m_{2}}T{\left(m_{1},m_{2}\right)}^{p}}$$

**FréchetChemNet Distance (FCD)** can predict the biological activity of drugs, as shown in Eq. (11).

(11) $$FCD\left(G,R\right)=||\mu_{G}-\mu_{R}|{|}^{2}+Tr(\sum G+\sum R-2{\left(\sum G\sum R\right)}^{\frac{1}{2}})$$

μ_*G*_ and μ_*R*_ are mean vectors, ∑*G* and ∑*R* are the covariance matrix of the penultimate layer activity on sets *G* and *R*.

**Tanimoto coefficient** based on molecular fingerprints is used to judge the degree of correlation between two data, as shown in Eq. (12).

(12) $$T\left(G,R\right)=\frac{\sum_{i}G_{i}\cap R_{i}}{\sum_{i}G_{i}\cup R_{i}}$$

**Scaffold similarity (Scaff)** is the cosine distance between the frequency vectors of the molecular scaffold as in Eq. (13).

(13) $$Scaff\left(G,R\right)=1-\text{cos}\left(S_{G},S_{R}\right)$$

*S*_*G*_ and *S*_*R*_ represent the frequency of the scaffold in molecule *G* and molecule *R*.

The above six indicators comprehensively examine the characteristics of the generated molecules. In order to quantitatively compare the distribution of the generated set and the test set, we use the following three auxiliary indicators:

**Molecular weight (MW)**: It is the sum of the atomic weights in the molecule.

**logP:** It reflects the distribution of a substance in oil and water. This value is the logarithmic value of the ratio of the partition coefficient between n-octanol and water. The larger the value of *logP* is, the more fat-soluble the substance is.

**Synthetics Accessibility Score (SA)**: It used to evaluate the difficulty of compound synthesis.

## Results and Discussion

### Composition of Datasets

The data set is divided based on Aliphatic Rings, Aromatic Rings, HBA, and HBD. The data set size is shown in Table 2, which shows the train size and test size of the molecules in the data set. In all experiments, we use the training set (X_train_ and Y_train_) to train and the test set (X_test_ and Y_test_) to evaluate the model.

**TABLE 2:** Structural transformations and dataset sizes.

Dataset	Aliphatic Rings	Aromatic Rings	HBA	HBD
X_train_	40,000	80,000	75,000	75,000
X_test_	69,682	18,220	37,149	37,149
Y_train_	40,000	80,000	75,000	75,000
Y_test_	10,329	53,717	10,764	12,785

**FIGURE 3:**
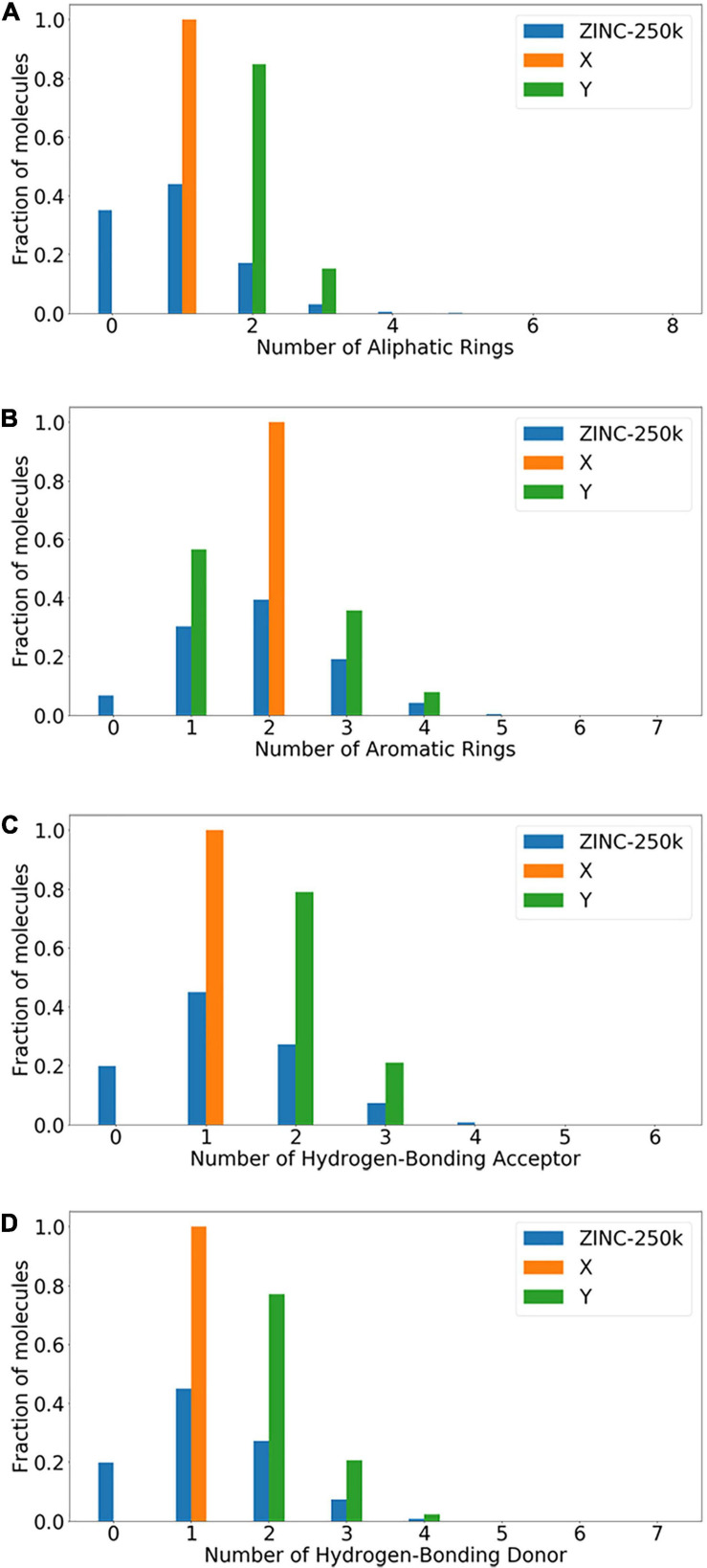
ZINC-250K data sets are distributed according to specified four characteristics. **(A)** Aliphatic Rings; **(B)** Aromatic Rings; **(C)** Hydrogen-Bonding Acceptor; **(D)** Hydrogen-Bonding Donor.

Figure 3 shows the experimental data set ratio distribution map in Table 2. The population of molecules with Aliphatic Rings, Aromatic Rings, HBA, and HBD and the distribution of X and Y are shown in Figure 3. In each histogram, the blue columns represent the distribution of the ZINC-250K data set according to the four characteristics. The orange bars represent the distribution of the X data set. The green bars represent the distribution of the Y data set.

### Structure Attributes

In the process of model training, due to the special structure of CycleGAN, the model will transform and reconstruct the structure of the molecular data on the two opposite regions X and Y. In Table 3, the structural performance and structural attributes of the model in the distribution of different characteristics are quantitatively computed, including Aliphatic Rings, Aromatic Rings, HBA, and HBD. A fully symmetrical structure is used in the model. When the model is constructing, we partly consider the problem of structural transformation. LA-CycleGAN model has been improved in terms of Success rate, Uniqueness and Non-identity as shown as the bolded data in Table 3. Aromatic Rings has the lowest conversion success rate and is the most difficult to convert. After optimization, Aliphatic Rings has the highest success rate for substructure conversion tasks. Under the distribution of HBA and HBD characteristics, the two data sets tend to be consistent in the direction of structural transformation. The success rate of HBA and HBD is only slightly different. Uniqueness has improved significantly in all distributions. This result shows that the LA-CycleGAN model can reduce the probability of repeated generation of molecules and avoid an increase in the overlap rate of molecules at the same region. During model verification, the Non-identity of HBA and HBD has been significantly improved after optimization. This result shows that the similarity of molecules has been improved with the conserved Aliphatic Rings. In summary, the LA-CycleGAN model presents the best ability to obtain data conversion over Mol-CycleGAN when training on a data set distributed according to Aliphatic Rings. It also proves that the data set is easy to change. It is easier to use in tests of drug production.

**TABLE 3:** Structure conversion assessment of generated molecules.

Model test		*X→*G*(*X*)*			*Y→*F*(*Y*)*		
Data	Model	Success rate	Uniqueness	Non-identity	Success rate	Uniqueness	Non-identity
Aliphatic Rings	Mol-CycleGAN	0.534	0.976	0.908	0.422	0.990	0.890
	LA-CycleGAN	**0.617**	0.982	**0.998**	**0.494**	0.991	**0.999**
Aromatic Rings	Mol-CycleGAN	0.535	0.986	0.908	0.421	0.995	0.889
	LA-CycleGAN	0.536	0.990	0.997	0.427	**0.999**	0.997
HBA	Mol-CycleGAN	0.608	0.987	0.697	0.378	0.987	0.684
	LA-CycleGAN	0.612	**0.996**	0.995	0.382	0.993	0.999
HBD	Mol-CycleGAN	0.602	0.985	0.658	0.380	0.988	0.648
	LA-CycleGAN	0.612	0.991	0.996	0.420	0.994	0.998

*The item with the highest data value in the column is indicated in bold.*

**FIGURE 4:**
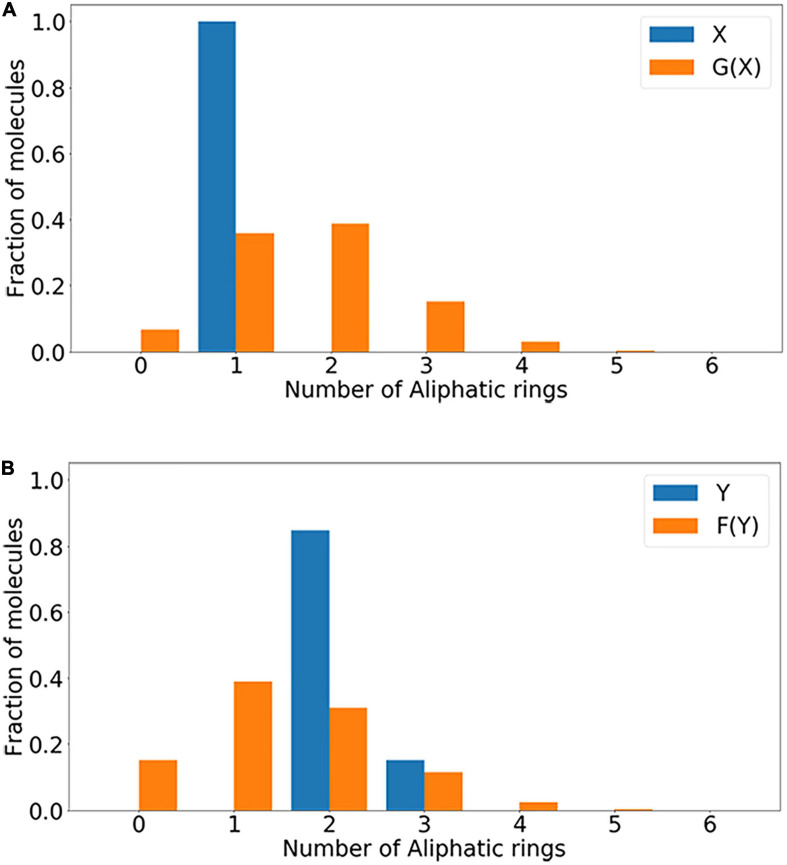
The distribution of Aliphatic Rings that generate molecules. **(A)** Distribution of the number of Aliphatic Rings in X and G(X); **(B)** distribution of the number of Aliphatic Rings in Y and F(Y).

As shown in Figure 4, in the distribution of Aliphatic Rings, without Aliphatic Ring molecules, there is only a difference in the number of rings between data sets X and Y. Therefore, the number of Aliphatic Rings in the newly generated molecular structure is significantly reduced, and the success rate of obtaining Aliphatic Ring conversion is higher. In the above-mentioned molecular data conversion processes based on the distribution of the characteristic element, molecules that do not contain the above-mentioned characteristic element are eliminated, which effectively improves the success rate of molecular transformation. At the same time, the number of molecules produced by the 3-ring Aliphatic Ring has increased significantly, and the number of Aliphatic Rings produced by the 1-ring and 2-ring Aliphatic Rings is still the largest as shown in Figure 4.

### Similarity Evaluation

**FIGURE 5:**
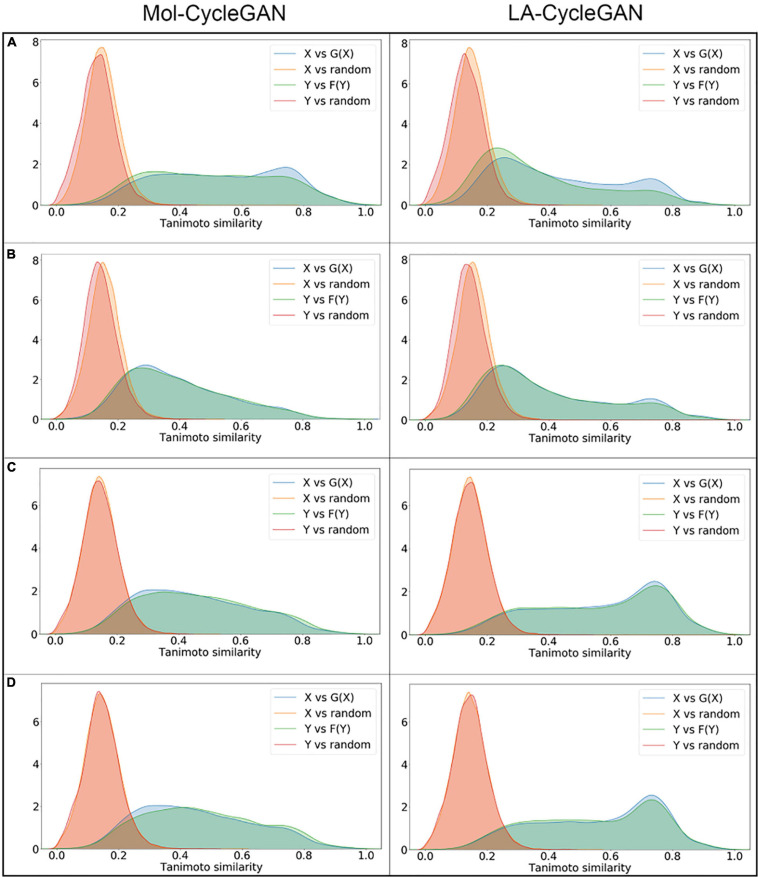
Density map of Tanimoto similarity between corresponding molecules. **(A)** Tanimoto similarity for the Aliphatic Rings subset; **(B)** Tanimoto similarity for the Aromatic Rings subset; **(C)** Tanimoto similarity for the HBA subset; **(D)** Tanimoto similarity for the HBD subset. The left panel is from Mol-CycleGAN and the right panel is from LA-CycleGAN.

In the chemical space, the similarity of molecular structure will directly affect the biological activity of similar molecules. In Figure 5, the Tanimoto similarity evaluation is performed on the data set of the distribution of various elements. It visually shows the similarity between each compound vector. Compared with Mol-CycleGAN, LA-CycleGAN achieve better Tanimoto similarity distribution between the generated molecules and the starting molecules. The Tanimoto similarity distribution also holds true for random molecules in the ZINC-250K data set. It can be seen that the molecular similarity of the LA-CycleGAN model is significantly increased. The Attention mechanism assigns different degrees of attention to different parts of the input data or feature map. Focused learning makes the weight distribution obtained in the process of molecule generation more concentrated, and can retain useful information. Finally, it will generate highly similar molecules. The distribution of similarity between the newly generated molecule and the starting molecule in the LA-CycleGAN model gradually tends to be consistent in the X and Y regions. Compared with the original model, the consistency of the similarity distribution of the optimized model has been adjusted and improved. To improve the similarity between two molecules, it is necessary to enhance the discrimination ability of the discriminator. Extract features from the input, and then add a dense layer of one-dimensional output to determine whether the extracted features belong to a specific category. The problem of judging true and false molecules is transformed into a binary classification problem. After convergence, the discriminator is used as a classifier for judging the true and false of molecular data.

Table 4 shows the prediction and verification of the model from the data structure of the four different feature distributions to determine the effectiveness of the model’s molecular generation. The Valid of the HBA distribution is the highest in the four different feature distributions. The Valid of the Aliphatic Rings distribution has not been changed. The effectiveness of the distribution of Aromatic Rings has increased the most. Novelty reveals the ability of the optimized model to generate new molecules. Aliphatic Rings generates the highest proportion of new molecules and the highest improvement. The LA-CycleGAN model has the highest IntDiv1 and IntDiv2 scores on the HBA distribution. The HBA molecule has the best performance in terms of generating internal diversity. The internal diversity of HBD distribution structure is slightly lower than that of HBA. It can be seen that the data structure based on the hydrogen bond system is easy to modify and reorganize molecules. Aromatic Rings has the most obvious performance in the Filters evaluation. As part of generating molecules, it can filter charged particles to satisfy the effectiveness of generated molecular compounds. At the same time, inhibiting the generation or deleting molecular fragments do not meet the designed expectations. In our evaluation, we divide the dataset according to the characteristics of the pharmacophore elements without considering the diversity of molecular skeletons. It would be an interesting study to compare the detailed effect of diversity of starting molecules on the diversity of generated molecule by finely tuning the degree of scaffold similarity in two sets X and Y (Benhenda, 2017; Gui et al., 2020).

**TABLE 4:** Performance evaluation of model on generating molecules.

Data	Model	Valid (↑)	Novelty (↑)	IntDiv_1_ (↑)	IntDiv_2_ (↑)	Filters (↑)
Aliphatic	Mol-CycleGAN	0.998	0.975	0.861	0.856	0.588
Rings	LA-CycleGAN	0.998	**0.992** ^a^	0.869	0.863	0.644
Aromatic	Mol-CycleGAN	0.996	0.988	0.867	0.861	0.615
Rings	LA-CycleGAN	0.998	0.988	0.868	0.872	**0.682**
HBA	Mol-CycleGAN	0.997	0.977	0.871	0.865	0.606
	LA-CycleGAN	**0.998**	0.987	**0.883**	**0.877**	0.674
HBD	Mol-CycleGAN	0.996	0.975	0.872	0.866	0.603
	LA-CycleGAN	0.998	0.987	0.883	0.866	0.673

*^*a*^The bolded data in Table 4 represents the item with the highest data value in the column.*

Table 5 shows the four evaluation indicators of FCD, SNN, Frag, and Scaff for the evaluation of the similarity of generated molecules. The chemical fragments generated by the model design have higher similarity and the optimized model has improved on these four evaluation indicators. Aliphatic Rings performs well in Fra, Scaff, and SNN. The fragments of the generated molecule and the starting molecule have the highest similarity ratio, and have a high degree of similarity in the direction of the chemical structure, so they have the same biological response. From an overall point of view, the performance of the LA-CycleGAN model has been comprehensively improved. LSTM solves the problem of gradient disappearance and improves the accuracy of molecule generation. The generated molecular data has a high proportion of scaffolds that are the same as the test set. Among the four data sets distributed according to characteristic elements, HBA has the most significant improvement and the highest proportion. FCD is the error function used to measure the activity of the resulting compound. In the optimized model, the result of Aromatic Rings achieves the smallest error and its biological activity is most similar to the starting molecule. In summary, from the four different chemical structure distributions, the model can generate pharmacophores with excellent biological activity.

**TABLE 5:** Evaluation of the similarity of the generated molecules.

Model test		FCD (↓)		SNN (↑)		Frag (↑)		Scaff (↑)	
Data	Model	Test	TestSF	Test	TestSF	Test	TestSF	Test	TestSF
Aliphatic Rings	Mol-CycleGAN	0.574	0.600	0.498	0.486	0.954	0.475	0.277	0.090
	LA-CycleGAN	0.568	0.600	**0.502** ^a^	**0.493**	**0.963**	**0.664**	0.374	0.108
Aromatic Rings	Mol-CycleGAN	0.391	0.047	0.142	0.467	0.142	0.109	0.563	0.157
	LA-CycleGAN	**0.361**	**0.046**	0.481	0.471	0.166	0.471	0.577	**0.199**
HBA	Mol-CycleGAN	0.389	0.442	0.479	0.464	0.331	0.473	0.759	0.134
	LA-CycleGAN	0.382	0.417	0.479	0.468	0.347	0.514	**0.799**	0.141
HBD	Mol-CycleGAN	0.392	0.444	0.480	0.464	0.318	0.456	0.660	0.137
	LA-CycleGAN	0.384	0.330	0.480	0.468	0.328	0.486	0.689	0.137

*^*a*^The bolded data in Table 5 represents the best value for the column.*

**FIGURE 6:**
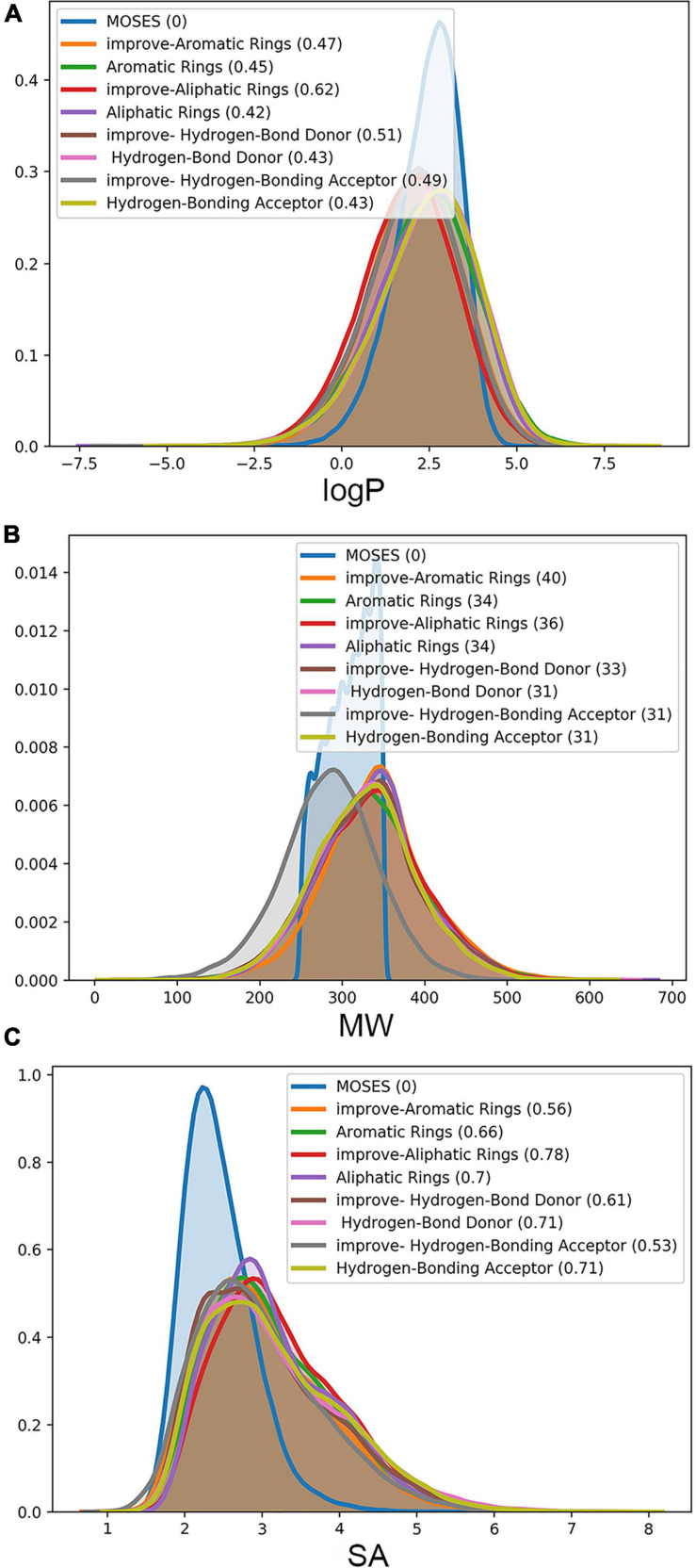
Attribute distribution of generated molecules. LA-CycleGAN model provides **(A)** the improved logP, **(B)** molecule weight (MW) and **(C)** Synthetics Accessibility (SA) Score.

We evaluate the generated molecules by using the evaluation indicators provided by MOSES. In Figure 6, the Mol-CycleGAN model and the LA-CycleGAN model are compared from LogP, MW, and SA. LogP shows that the concentration ratio of octanol to water in the LA-CycleGAN model has increased significantly. Compared with Mol-CycleGAN, our LA-CycleGAN model has reproduced the best LogP distribution for the set of Aliphatic Rings, reaching 0.62. The overall Mol-Weight has been improved, and the overall molecular weight has been increased. Based on the molecular weight of the generated and tested sets, it can be judged whether the generated set is biased toward lighter or heavier molecules. In MW plots, the MW of the generation set of the improved model has been increased, which means that the generated set of the new model is biased toward heavier molecules. SA reveals the difficulty of drug synthesis and is used to evaluate learning models. Aliphatic Rings is the most difficult to synthesize drugs. The difficulty of synthesizing has decreased for the other three groups of compounds. For example, the SA decreases from 0.71 to 0.53 for the set of HBA.

**FIGURE 7:**
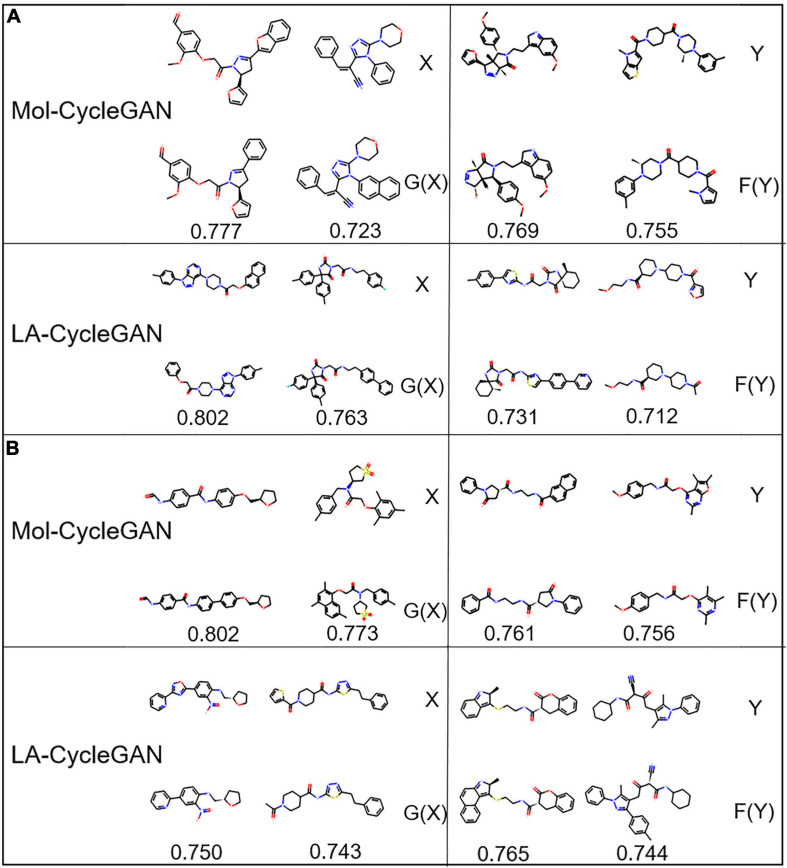
Molecular structure diagram. **(A)** Molecules from the aliphatic ring subset; **(B)** molecules from aromatic ring subset. In each sub-figure, the upper layer shows the starting molecules, the middle layer shows the generated molecules, and the bottom layer shows the similarity score.

**FIGURE 8:**
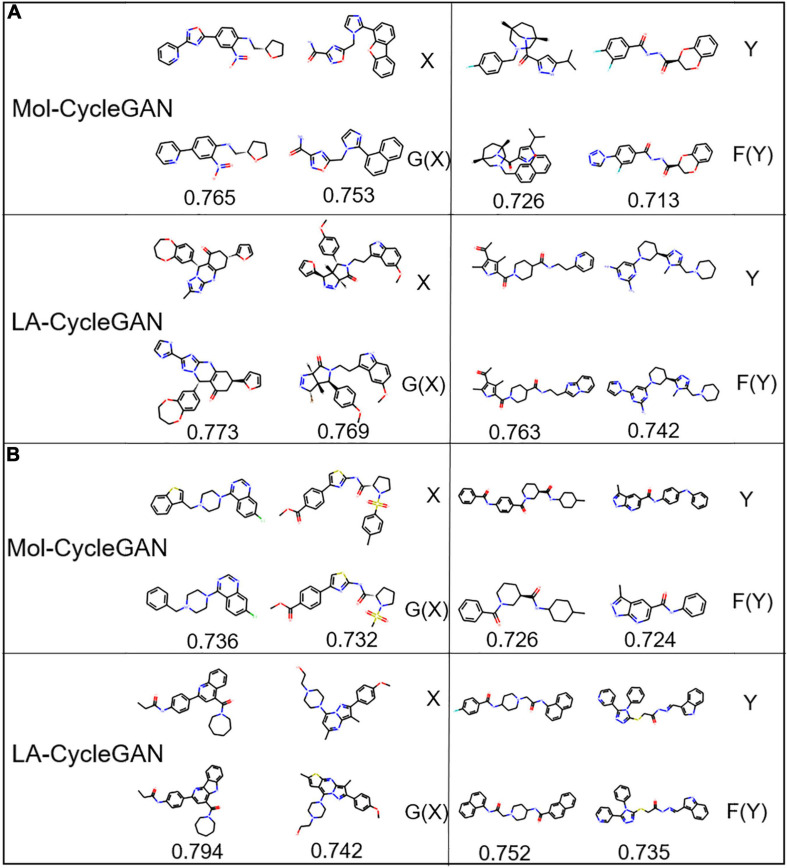
Molecular structure diagram. **(A)** Molecules from the HBA subset; **(B)** molecules from HBD subset. In each sub-figure, the upper layer shows the starting molecules, the middle layer shows the generated molecules, and the bottom layer shows the similarity score.

The visualization process of the Mol-CycleGAN and LA-CycleGAN model to generate molecules in the four data distributions is shown in Figures 7, 8. As shown in molecular diagram, the obtained molecules are all effective molecules. For both models, the similarity of X and G(X) is generally higher than that of Y and F(Y). LA-CycleGAN displays comparative performance as the Mol-CycleGAN. Especially in the task of reproducing molecules of HBA and HBD, LA-CycleGAN produces higher similarity score than the Mol-CycleGAN as shown in Figure 8. After optimizing the model, HBD obtained the highest similarity score, indicating that the similarity has been improved significantly.

## Conclusion

We proposed LA-CycleGAN as a new method of molecule generation by embedding LSTM and Attention mechanism in the CycleGAN model architecture. LSTM and Attention mechanisms are introduced to solve the problem of gradient disappearance and improve the accuracy of molecule generation. The generator in the CycleGAN uses Autoencoder to map the molecular structure into the latent chemical space. The decoder returns the sampled potential chemical space to the original space. Finally, the molecular map is obtained from the potential space of JT-VAE. The proposed model is evaluated by four subsets with different feature distributions extracted from the ZINC-250K dataset. The generated molecules between the Mol-CycleGAN model and the LA-CycleGAN model are quantitatively evaluated. The experimental results show that the similarity and success rate of molecules generated by the LA-CycleGAN model have been significantly improved over Mol-CycleGAN. The generated molecules have similar or even the same biological activity as the starting molecules. LA-CycleGAN model can act as one of molecule generation method to generate molecules with similar drug-like compounds. The attention-based deep neural network can be interpreted by furfure analyzing the relationship between the attention scores of features and the expected generated molecules. It would be an attractive work to make *de novo* molecular generation model interpretable in future study.

## Data Availability Statement

The datasets presented in this study can be found in online repositories. The names of the repository/repositories and accession number(s) can be found below: https://osf.io/42x7d/?view_only=c2cf708215804280bdf24c2bfb4f690b, Open Science Framework.

## Author Contributions

FW, LiX, and SC derived the concept. FW and XF wrote relevant code and performed the experimental work and analysis. XF, XG, and LeX wrote the original manuscript. FW, XF, XG, and LeX provided the feedback and critical input. LiX and SC prepared the revised version of the manuscript. All authors read and approved the final manuscript.

## Conflict of Interest

The authors declare that the research was conducted in the absence of any commercial or financial relationships that could be construed as a potential conflict of interest.

## Publisher’s Note

All claims expressed in this article are solely those of the authors and do not necessarily represent those of their affiliated organizations, or those of the publisher, the editors and the reviewers. Any product that may be evaluated in this article, or claim that may be made by its manufacturer, is not guaranteed or endorsed by the publisher.

## Abbreviations

GAN: Generative Adversarial Networks
DL: Deep Learning
VAE: variational autoencoder
JT-VAE: Junction Tree Variational Autoencoder
LSTM: Long Short-Term Memory
CBDD: Computer-aided drug Design
RNN: recurrent neural network.
